# Electroanalysis of Naringin at Electroactivated Pencil Graphite Electrode for the Assessment of Polyphenolics with Intermediate Antioxidant Power

**DOI:** 10.3390/antiox11122306

**Published:** 2022-11-22

**Authors:** Iulia Gabriela David, Simona Carmen Litescu, Raluca Moraru, Camelia Albu, Mihaela Buleandra, Dana Elena Popa, Sorin Riga, Adela Magdalena Ciobanu, Hassan Noor

**Affiliations:** 1 Department of Analytical Chemistry and Physical Chemistry, Faculty of Chemistry, University of Bucharest, Panduri Av. 90-92, District 5, 050663 Bucharest, Romania; 2 National Institute of Research and Development for Biological Sciences, 296 Independenței Bd., District 6, 060031 Bucharest, Romania; 3 Department of Stress Research & Prophylaxis “Prof. Dr. Al. Obregia” Clinical Hospital of Psychiatry, Berceni Av. 10, District 4, 041914 Bucharest, Romania; 4 Department of Psychiatry “Prof. Dr. Al. Obregia” Clinical Hospital of Psychiatry, Berceni Av. 10, District 4, 041914 Bucharest, Romania; 5 Discipline of Psychiatry, Neurosciences Department, Faculty of Medicine, “Carol Davila” University of Medicine and Pharmacy, Dionisie Lupu Street 37, 020021 Bucharest, Romania; 6 Department of Surgery, Faculty of Medicine, “Lucian Blaga” University Sibiu, Lucian Blaga Street 25, 550169 Sibiu, Romania

**Keywords:** naringin, pencil graphite electrode, total content of polyphenolics, intermediate antioxidant power, disposable, electroactivated electrode, grapefruit

## Abstract

A simple and rapid differential pulse voltammetric (DPV) method using a single-use electroactivated pencil graphite electrode (PGE*) is proposed for the rapid screening of the total content of polyphenolics (TCP) with intermediate antioxidant power (AOP) in grapefruit peel and fresh juice. The results were compared and correlated with those provided by the HPLC-DAD-MS method. NG voltammetric behavior at PGE* was studied by cyclic voltammetry and an oxidation mechanism was suggested. The experimental conditions (type of PGE, electroactivation procedure, pH, nature and concentration of supporting electrolyte) for NG DPV determination were optimized. The NG peak current varied linearly with the concentration in the ranges 1.40 × 10^−6^–2.00 × 10^−5^ and 2.00 × 10^−5^–1.40 × 10^−4^ mol/L NG and a limit of detection (LoD) of 6.02 × 10^−7^ mol/L NG was attained. The method repeatability expressed as relative standard deviation was 7.62% for the concentration level of 2.00 × 10^−6^ mol/L NG. After accumulation for 240 s of NG at PGE* the LoD was lowered to 1.35 × 10^−7^ mol/L NG, the linear range being 6.00 × 10^−7^–8.00 × 10^−6^ mol/L NG. The developed electrochemical system was successfully tested on real samples and proved to be a cost-effective tool for the simple estimation of the TCP with intermediate AOP in citrus fruits.

## 1. Introduction

**Figure 1:**
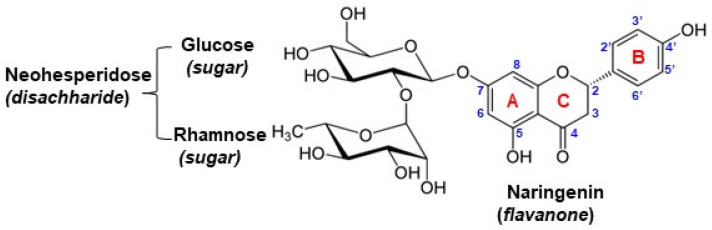
Chemical structure of naringin (NG).

Naringin (NG) (4′,5,7—trihydroxy flavanone 7—rhamnoglucoside), a natural plant phenolic, is a flavanone-7-O-glycoside formed between the aglycon naringenin and the disaccharide neohesperidose, composed of a glucose and a rhamnose subunits (Figure 1). Its structure was explained for the first time in 1928 [1]. NG is mainly extracted from grapes and citrus fruits [2], the highest concentrations being found in the peel of grapefruit, *Citrus paradisi* (3.25%) and bitter orange, *Citrus aurantium* (2.11%) conferring them the bitter taste [1]. Studies revealed that the NG levels in plants decrease with their development towards maturity due to the conversion of this bioflavonoid into non-bitter compounds such as aglycone naringenin (NGN) [3]. NG was also detected in dried rosemary leaves (1261 mg/kg), in certain orchid-type leaves (110 mg/kg), peppermint (20 mg/kg), tomatoes (0.88 mg/kg) and other herb species [1].

The increased interest for studying NG is based on the wide spectrum of its health benefits [2,4] due to its antioxidant, anti-inflammatory, bactericidal [5], anti-cancer, antimutagenic, cholesterol-lowering, and neuro- and cardiovascular-protective effects [6,7]. The presence of the –OH groups in the NG structure confers antioxidant properties to this bioflavonoid, but at higher concentrations, it exhibits pro-oxidant activities [8]. It was shown that NG reduces DNA damage by controlling the production of free radicals, with its radical-scavenging activity being dose-dependent [9]. The antitumoral activity of this phyto-compound in various cancers implies multiple mechanisms, some of them relying on its property to eliminate free radicals [10]. Due to its antioxidant and anti-inflammatory effects, NG alone [11] or in combination with trimetazidine [12] may exert protective activity against renal damage. Repeated administration of NG in mice induced anxiolytic-, antidepressant- and antiepileptic-like effects and increased locomotor activity, cognitive and memory performance via mechanisms including the enhancement of the antioxidant defense systems, the inhibition of lipid peroxidation, nitrosative stress and neuroinflammatory processes [13]. Studies revealed NG’s therapeutic effects in common musculoskeletal pathologies such as osteolytic and degenerative joint diseases, bones and joint infections [14,15]. Based on its ability to regulate the degree of reactive oxygen species, NG can help wound healing by favoring tissue regeneration [16]. Long-term consumption of NG presented no toxic effects on rats and humans, but after oral administration, it is poorly absorbed in the blood circulatory system due to its low bioavailability [15] which is caused by its reduced water solubility and permeability. Therefore, NG remains for a certain period in the gastrointestinal tract, where it is transformed into its main metabolite, NGN [17].

Considering all the above-listed beneficial effects of NG on human health, which implies the importance of its daily intake from natural sources, mainly from citrus fruits, it is of interest to develop simple, fast and reliable methods for the detection of this bioflavanone and the quality control of its content or of the total antioxidants content in real samples. Various natural phenolics, some of them with very similar structures (e.g., flavanones and other flavonoids), coexist in different parts of the plants, and therefore, usually before the actual determination, the extraction [18,19] and/or separation [20,21] of NG from the sample matrix (e.g., plants, plant extracts, food, juices, etc.) was necessary. A recent review presents an overview of the extraction and sample preparation methods for NG chromatographic analysis from citrus fruits [1]. NG is a chiral compound and the ratio of the optically active isomers depends on the fruit maturation state [14]. Therefore, the chiral determination of NG from citrus peel and pulp was performed by ultra-performance liquid chromatography (LC) tandem mass spectrometry (MS) [22], while rapid resolution LC-MS coupled with isotope deuterium labeling was applied to the simultaneous quantification of NG and its metabolites NGN and 3-(4′-hydroxyphenyl) propanoic acid [17]. The determination of flavonoids, among them NG, from fruits and juices was also carried out by capillary electrophoresis with a diode array [20] or electrochemical detection using bare [23] or modified electrodes [24]. These techniques are selective and sensitive, but they are also expensive and time-consuming.

Electroanalysis is a simpler, more rapid and cost-effective alternative for antioxidants and, consequently, NG determination, having the additional advantage that the instrumentation can be easily miniaturized and used for the assessment of small sample volumes. However, despite its electroactivity conferred by the –OH groups, NG is one of the flavonoids which was relatively little studied by electrochemical methods. A recent paper describes the use of an ion-exchange-based resistive sensor for the NG quantification between 1.72 × 10^−6^ and 1.72 × 10^−4^ mol/L and its removal from citrus juice [25]. An interdigitated electrode-based conductive sensor connected to a microcontroller platform, presenting a linear range of 25–100 ppm NG was applied to monitor the maturity stage of pomelo fruits by assessing their NG content [3].

More selective electrochemical techniques are the voltammetric ones and the possibility of employing various modified electrodes enables the enhancement of both their sensitivity and selectivity. A thorough literature research pointed out that starting from 1998 till now there are relatively few studies regarding the electrochemical behavior and (volt)amperometric determination of NG [8,26,27,28,29,30,31], two of them being based on photoelectrochemistry [6,32]. Voltammetric techniques constitute also an important tool in the investigation of interactions between different bioactive molecules. NG square wave voltammetry with a carbon paste electrode was used to examine the NG interaction with DNA by monitoring the changes in the position and intensity of the adenine and guanine signals from oligonucleotides and dsDNA. The study revealed that at concentrations up to 1.00 × 10^−5^ mol/L (which is more than two times higher than the NG dose absorbable in an average human), NG acts as an antioxidant, having a genoprotective role, while above this level it could have pro-oxidant activity, favoring DNA oxidation [33]. One major drawback of the voltammetric methods is the fouling/passivation of the working electrode surface during measurements, e.g., by the formation of polymeric films, as is the case in the voltammetric analysis of polyphenols [34]. Consequently, in order to obtain reproducible results, the regeneration of the sensors’ electroactive surface area is compulsory. This involves an additional, tedious and time-consuming step, which can be eliminated by using disposable electrodes such as the pencil graphite electrode (PGE). In recent years PGE gained increasing applicability due to its similar or even better electrochemical characteristics in comparison to other commonly used working electrodes, besides its other inherent economic advantages (cost-effectiveness and easy availability) [35,36]. However, there are few reports using this electrode for the electroanalysis of bioflavonoids [34,37,38,39,40,41] which, based on their oxidation potential [42], are considered to have intermediate antioxidant power (AOP) [43]. To the best of our knowledge, there is no research that has been conducted on the NG electroanalysis of disposable, bare PGE. The results from this study for NG voltammetric quantification are comparable with some previously reported at other electrodes, with better results being obtained using chemically modified electrodes, as was expected. However, the favorable features of the PGE make it a versatile tool for the rapid and reliable screening of the total content of polyphenolics (TCP) with intermediate AOP of citrus fruits and their derivatives (e.g., juices).

## 2. Materials and Methods

### 2.1. Instrumentation

Voltammetric measurements have been performed on an Autolab PGSTAT 12 electrochemical system connected to a PC running the GPES4.9 software. A voltammetric cell containing a Ag/AgCl, KCl (3M) reference electrode, a Pt counter electrode and a pencil graphite electrode (PGE), if not stated otherwise, was the working electrode.

The tested working electrodes were different hardness, with PGEs of 0.50 mm graphite pencil leads (B, HB and 2B from the brands Laco and Rotring) having the geometrical active surface area (A_g_) of 0.1589 cm^2^; a glassy carbon electrode (GCE) and a Pt electrode with the A_g_ of 0.0700 cm^2^ and 0.0314 cm^2^, respectively. Nevertheless, the main working electrode was the PGE, which consisted of 6.00 cm long, commercially available graphite pencil leads cut in half and introduced with the cut edge into a mechanical pencil lead used as a holder; 1.50 cm of the lead remained outside and always only 1.00 cm of this part was immersed in the analyzed solution so that the A_g_ of the electrode was always constant [41]. The electrical contact was realized by a copper wire soldered on the metallic part of the pencil. A new graphite pencil lead was employed for each measurement, excepting the studies requiring the recording of repetitive cyclic voltammograms.

In order to assure the reproducibility of the other solid electrode surface, the GCE and the Pt electrode were polished with 0.05 µm alumina powder on a special material, then rinsed with distilled water and air dried.

A pH/mV-meter Consort P901 Scientific Instrument (Belgium) equipped with a pH-sensitive combined glass electrode was used to measure the solutions’ pH.

Chromatographic analysis has been performed on a Shimadzu 20AD instrument with the following components: LC-20AD SP Shimadzu pumps with a maximum pressure of 400 bar, Shimadzu degasser DGU-20A5-Degasser, automatic injector, detector Shimadzu SPD-M20A Diode Array Detector, CTO-20AC Column Oven (thermostatic compartment of the column), LCsolution software. The separation was carried out on a chromatographic column of the Kromasil C18 type, 5 μm, 150 × 4.6 mm.

### 2.2. Reagents and Solutions

Naringin (NG) (≥95.0%, HPLC), naringenin (NGN) (>95%, p.a.), caffeic acid (≥98.0%, HPLC), gallic acid monohydrate (≥98.0%, ACS reagent), ethanol (≥99.5%, ACS reagent), potassium phthalate monobasic (KHPT) (ACS reagent), H_3_BO_3_ (1 g/tablet), CH_3_COOH (≥97%, ACS reagent), H_3_PO_4_ (≥85 wt. % in water, ACS reagent), Na_2_HPO_4_ × 2H_2_O and KH_2_PO_4_ (p.a., ACS reagent), NaOH (pellets), K_3_Fe(CN)_6_ (≥99.0%, ACS reagent) and KCl (99.0–100.5%, ACS reagent) were purchased from Sigma-Aldrich (Munich, Germany).

The 5.00 × 10^−3^ mol/L NG stock solution was daily prepared by dissolving the proper, accurate weighted amount of NG and diluting it with ethanol to the mark of a 10 mL volumetric flask. When not in use, this solution was refrigerated. The working solutions were obtained in 10 mL volumetric flasks by successive dilutions of the stock solution using an adequate supporting electrolyte. Acetate buffer solution (ABS; pH 3.80; 4.00 and 4.60), phosphate buffer solution (PBS; pH 7.00) and 0.05 mol/L (if not specified another concentration) KHPT (pH 4.00) were employed as supporting electrolytes. Britton–Robinson buffer (BRB) solutions with a pH between 1.81 and 11.20 were chosen for the investigation of the pH influence on NG voltammetric behavior. All aqueous solutions were prepared with deionized water.

### 2.3. Procedures

Cyclic voltammetry (CV) was applied to investigate NG voltammetric behavior, while differential pulse voltammetry (DPV) was used to establish the optimum conditions for its quantitative determination. The DPV peak currents were measured after the baseline correction was applied.

### 2.4. Real Samples Analysis

The applicability of the developed analytical tool on NG assessment in real samples was performed for pink and yellow grapefruit, being analyzed its peel and the fresh juice. Samples to be analyzed were obtained as follows: 40 (±0.026) g of vegetal material (fruit or peel) were weighed and further used for NG extraction. The peel was subjected to ethanolic extraction (100 mL) for 2 h, under continuous shaking, while fruits were squeezed, then a certain volume (20 mL) of juice was mixed with an equal volume of ethanol, shaken for 20 min for NG extraction. Prior to the voltammetric analysis, juice samples were filtered and diluted with supporting electrolyte (0.05 mol/L KHPT pH 4.00) to reduce matrix effects and bring the TCP within the linear range of the previously established voltammetric method for NG and NGN. HPLC-DAD-MS analysis of the alcoholic pectin precipitation from the fresh juice was carefully performed; the sample was filtered and centrifuged; the supernatant was used for analysis.

When determining NG content from juices, the standard addition method was used. 1.5 mL of NG standard solution of three different concentrations was added to the same volume of sample, and the DPV measurements were performed. The sample without any NG addition was also analyzed. The NG content was assessed using the values of the peak intensities. Three replicates were analyzed for each sample.

## 3. Results and Discussion

### 3.1. Establishing the Optimum Conditions for NG Voltammetric Analysis

#### 3.1.1. The Influence of the Working Electrode Surface

**Figure 2:**
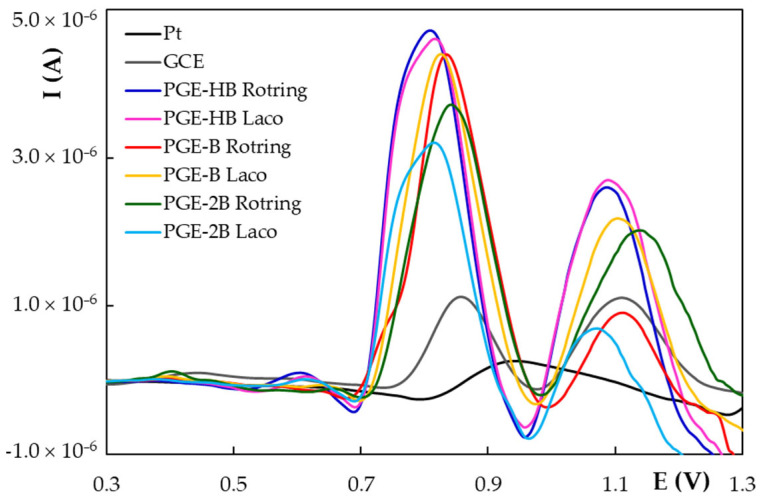
DP voltammograms recorded at different working electrodes for 1.80 × 10^−5^ mol/L NG in ABS pH 4.00 solution.

Due to the fact that the material representing the electroactive surface of the working electrode has a major effect on the electrochemical behavior of any analyte, this investigation started with the study of NG voltammetric response on various unmodified electrodes, namely the GCE, Pt electrode and PGEs constituted of graphite leads with different hardness, from two different manufacturers. Pencil graphite leads are composite materials and their hardness depends on the graphite/clay (binder) ratio, those with a higher content of graphite (the B types) being softer and with a higher degree of blackness (the 2B types) [36]. DPVs recorded for NG on the above-mentioned working electrodes (Figure 2) emphasized at the Pt electrode only one large, less intense peak at 0.940 V. Two anodic signals, a sharp and clearly defined one with the peak potential (E_pa1_) around 0.800 V and a smaller one situated at about 1.100 V, was distinguished at carbon-based electrodes. NG oxidation signals observed at GCE are well-defined but less intense than those recorded at any type of PGE. This outcome can be correlated with the geometrical active surface area of the tested working electrodes, which increased as follows: Pt (0.0314 cm^2^) < GCE (0.0700 cm^2^) < PGE (0.1589 cm^2^). The highest NG anodic peaks were obtained on the HB-type PGEs, independent of the manufacturer. However, the peak current depends also on the analyte concentration. Therefore, the sensitivities (S, A × L/cm^2^ × mol) of the tested electrodes were calculated in order to characterize the electrochemical properties of the electrode material towards NG and they varied in the order: Pt (0.92) < GCE (0.98) < PGE_2B_Laco (1.30) < PGE-2B_Rotring (1.38) < PGE_B_Laco = PGE-B_Rotring (1.65) < PGE_HB_Laco (1.79) < PGE-HB_Rotring (1.86). The Rotring HB type PGE was employed in further investigations due to the fact that it presented both the highest sensitivity for NG oxidation and the best mechanical rigidity.

The sensitivity and selectivity of an electrochemical method could be improved by modifying the electroactive surface of the working electrode. The simplest way to change the surface of carbon-based electrodes is electrochemical pretreatment [44,45]. Data from the literature showed that the efficiency of this procedure depends both on the conditions under which it is carried out and on the analyte [35]. Thus, the Rotring HB type PGEs were electrochemically pretreated both potentiodynamically (by CV) and potentiostatically using four different types of supporting electrolytes and the currents of the NG main anodic signal obtained by DPV (E_p_ ~0.800 V) were compared with that recorded at non-pretreated Rotring HB type PGEs. The results emphasized (Table 1) that the highest NG oxidation peaks were obtained at the electrodes activated by CV in NaOH solution and at a constant potential in PBS pH 7.00. However, the voltammograms recorded at the PGE activated potentiodynamically in an alkaline medium were very noisy; therefore, Rotring HB type PGEs pretreated potentiostatically (2.00 V; 60s) in neutral PBS solution, noted further as HB_PGE*, were used in all subsequent investigations.

According to the literature data, besides the formation of oxygen-containing groups, another reason for the peak current enhancement by the electrochemical pretreatment of carbon-based electrodes is the increase in the effective surface area due to the produced porosity [46]. Using the Randles–Sevcik equation and the results of CV measurements performed at bare HB type PGE and HB_PGE* in 5.00 × 10^−4^ mol/L K_3_Fe(CN)_6_ in 0.50 mol/L KCl (data not shown) the potentiostatic activation of HB type PGE in PBS pH 7.00 medium resulted in a ~21% increase in the working electrode effective surface area.

#### 3.1.2. The Influence of the pH and Nature of the Supporting Electrolyte

**Figure 3:**
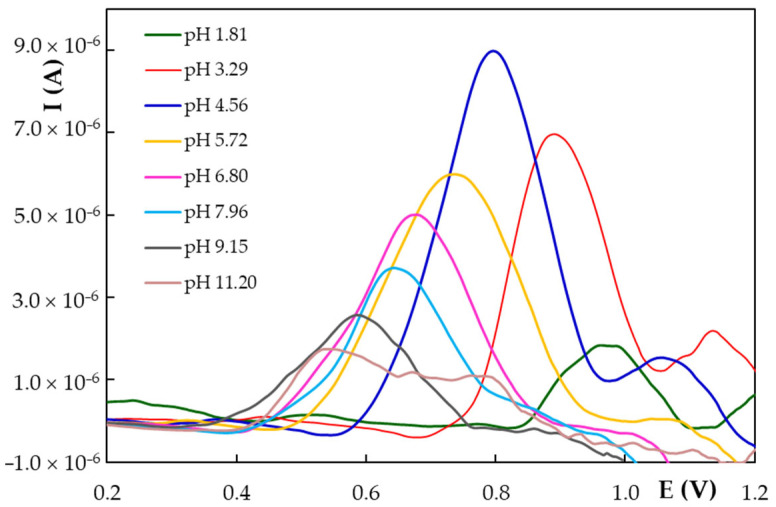
Selected DP voltammograms recorded at HB_PGE* for 7.00 × 10^−5^ mol/L NG in BRB solutions with different pH values.

The pH and nature of the supporting electrolyte play an essential role in the voltammetric behavior of the analytes, especially of those with ionizable functional moieties as is the case for NG, which contains several -OH groups in its structure (Figure 1). A study of the pH influence is highly significant even for the potential antioxidant activity of the NG, due to the fact that pH is influencing even the active compound mesomeric structure. It is known that H^+^ ionization from phenolic-OH, especially in protic solvents, is associated with antioxidant activity by hydrogen transfer. The effect of the solution pH on the voltammetric response of NG was investigated by CV (Figure S1) and DPV (Figure 3) using supporting electrolyte BRB solutions with pH values in the range of 1.81 to 11.20. Both voltammetric techniques emphasized that the peak current and potential are influenced by the solution pH. Both CV and DPV recordings showed that the position of NG anodic peaks moved towards less positive potentials with pH increasing, suggesting that NG electrode processes involved protons besides electrons. There was a linear dependence between the potential of the NG main anodic signal and the solution pH. Comparing the slopes of the regression equations describing the E_p_ = f(pH) dependencies obtained by CV and DPV (Table 2) with the theoretical slope of 0.059 x/n V from the Nernst relation (x represents the number of protons and n the number of electrons involved in the oxidation process generating the signal), the ratio x/n was assessed to be approximately 1.

Cyclic and DP voltammograms emphasized that the NG main anodic peak was highest at pH 4.56. According to data previously reported in the literature [8], the decrease in the peak currents with the increase in the solution pH is a characteristic of natural polyphenolics and may be due to their oxidation by atmospheric oxygen, which is favored in alkaline media. Data regarding the ~1 electron exchange and the NG structure in acidic media are supporting the idea that NG, as a participant in an antioxidant/free radicals scavenging mechanism type “electron transfer plus H transfer” is an active antioxidant in lipoperoxidation and reducing hydroperoxides formation.

Due to the fact that DPV is more sensitive than CV and NG oxidation signal from less positive potentials was the highest, these two were exploited in further investigations. The effect of the supporting electrolyte on NG main voltammetric signal at HB_PGE* was tested using various solutions (BRB, ABS and KHPT) with pH in the range of 3.80 to 4.60 (Figure S2). The KHPT concentration in the solution with pH 4.00 also varied between 0.025 and 0.20 mol/L (Figure S3); 0.05 mol/L KHPT pH 4.00 solution was selected to be used for the subsequent studies because in this medium the NG DPV anodic signal at HB_PGE* was best shaped, having the highest currents.

### 3.2. Voltammetric Behavior of NG at HB_PGE*

**Figure 4:**
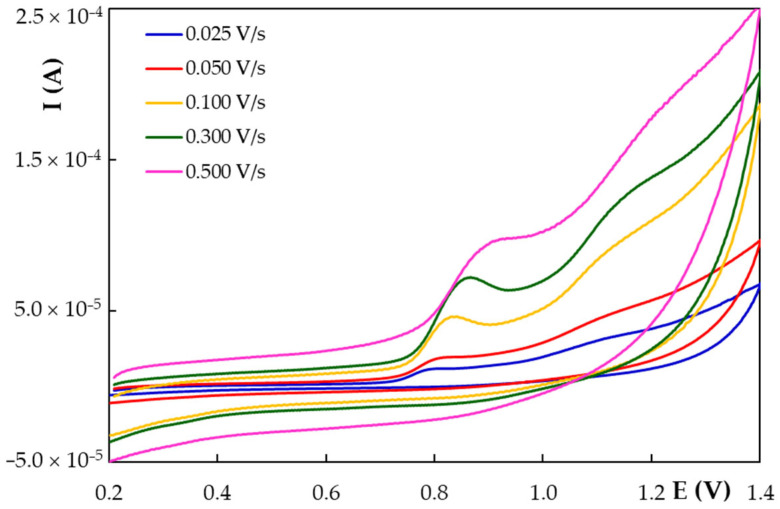
Cyclic voltammograms recorded with different scan rates at HB_PGE* for 1.00 × 10^−3^ mol/L NG in 0.05 mol/L KHPT pH 4.00 solution.

Cyclic voltammetry is the technique of choice for investigating the electrochemical behavior of an analyte. Therefore, cyclic voltammograms were recorded for NG at HB_PGE* applying different scan rates (v) ranging from 0.025 to 0.500 V/s (Figure 4). One can see that NG presented a well-defined anodic signal followed by another, less-defined wave, situated at more positive potentials and no reduction peak, emphasizing the irreversibility of NG oxidation at HB_PGE*. It is also obvious that by applying higher potential sweep rates the peak currents increased and the potentials shifted towards more positive values, confirming the NG irreversible electrode process. The regression equations of the I_p_ = f(v) and I_p_ = f(v^1/2^) dependencies (Table 3) showed good linearity, indicating that the electrode process generating NG main oxidation signal was controlled by the analyte adsorption onto the HB_PGE* surface, as well as by the NG diffusion towards the working electrode. The slope (0.8345) of the log I_p_ = f(log v) (Table 3), situated between 0.5 and 1.0, which are the theoretical values for diffusion and adsorption-controlled processes, respectively, confirmed the mixed nature of NG oxidation at HB_PGE* [47].

**Figure 5:**
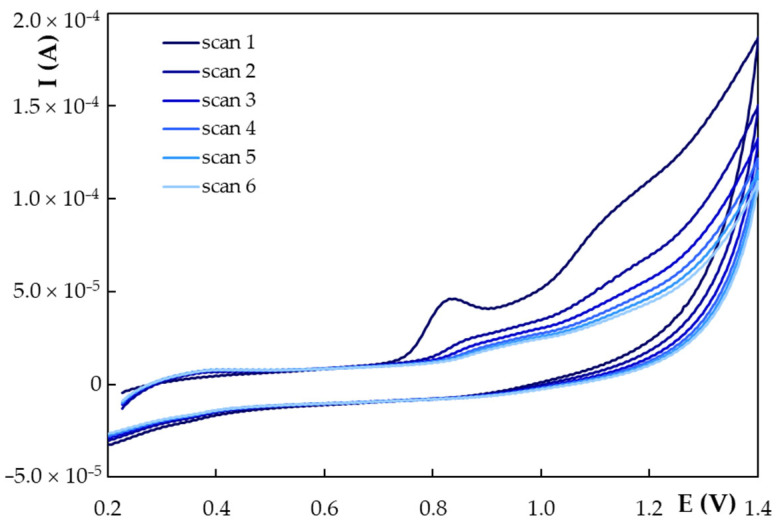
Repetitive cyclic voltammograms recorded at HB_PGE* for 1.00 × 10^−3^ mol/L NG in 0.05 mol/L KHPT pH 4.00 solution at a scan rate of 0.100 V/s.

Considering the Laviron relation [48] (Equation (1)) which describes the E_p_ variation with the potential scan rate for an irreversible electrode one can assess the number of exchanged electrons (*n*).

(1) $$E_{p}=E^{0}\prime +\frac{0.0591}{\alpha \times n}log\frac{0.0257\times k^{0}}{\alpha \times n}+\frac{0.0591}{\alpha \times n}logv\;$$

where *E*^0′^ represents the formal redox potential (V), α the charge transfer coefficient and *k*^0^, the standard heterogeneous rate constant of the reaction (s^−1^). The formal redox potential (*E*^0′^, V), assessed from the intercept of the *E_p_* = f(*v*) plot [49] (Table 3), was found to be 0.7544 V. Further, knowing *E*^0′^ and the intercept of the *E_p_* = f(log *v*) the standard heterogeneous rate constant was calculated to be 494.40 s^−1^. Comparing the slope of the *E_p_* = f(log *v*) regression equation (Table 3) with that of 0.0591 from Equation (1) and considering that the value of the charge transfer coefficient (α) is 0.5 for an irreversible electrode process [50], the number of electrons (*n*) involved in NG oxidation, generating its main anodic signal, was calculated to be 1.26, which can be approximated to *n* = 1.00. Considering these results and the fact that according to Brodowska et al. [51] the hydroxyl group from position 4′ in ring B (pK_a4′_ 8.90) is more acidic than the moiety from position 5 in ring A (pK_a5_ 11.85) (Figure 1), one can consider that NG irreversible oxidation involving one electron and one proton generates a radical at the hydroxyl group from ring B. This finding is in agreement with data previously reported in the literature by Ziyatdinova et al. [26] and with the theoretical results obtained by Milicevic and Novak Jovanovic [52] using quantum chemistry computation models, according to which at low pH values the flavonoids electrooxidation involves the extraction of an electron followed by the release of a proton (eH mechanism) yielding a semiquinone radical. The formed radical can then participate in dimerization and polymerization reactions, this fact being supported by the decrease in the anodic peak current with increasing the number of repetitive cyclic voltammetric scans recorded at the same working electrode (Figure 5). The height of the NG main oxidation peak was reduced by about 82% in the second scan and approximately 93% in the third scan with respect to the one obtained in the first direct scan, while it remained almost constant in the next scans. This behavior suggested the possible formation of a non-conductive polymer passivating the electrode surface, hindering thus the electron transfer, which resulted in the decrease in the peak current.

The nature of the second NG anodic peak was not investigated, but considering the data reported by Ziyatidinova et al. [28], it could be attributed to the oxidation of the hydroxyl group grafted on ring A.

### 3.3. Quantitative Determination of NG at HB_PGE*

#### 3.3.1. The Stability of NG Stock and Working Solutions

NG is a bioflavonoid with antioxidant properties and thus it is predictable that in solution it may be oxidized in atmospheric conditions. Due to the fact that the stock solution was used for the preparation over time of diluted solutions and, on the other hand, voltammetry has the advantage of performing several measurements on the same working solution which is preserved in ambient conditions, the stabilities of the stock (5.00 × 10^−3^ mol/L NG in ethanol) and working solutions (8.00 × 10^−6^ mol/L NG in 0.05 mol/L KHPT pH 4.00) were tested. To establish how long a stock solution can be used, the DPV currents of the NG main anodic signal were measured for working solutions freshly obtained from a fresh stock solution or from stock solutions maintained for certain periods of time (1 h, 2 h, 4 h, 1, 2, 4 and 7 days) either in the refrigerator or in ambient conditions. The results emphasized that the peak current remained almost constant if the working solution was employed on the day of preparation, while starting with the next day, higher oxidation signals were obtained at the same potential, regardless of the storing conditions. Thus, in order to ensure reliable results, the NG stock solution was always prepared on the day of its utilization and when not in use it was kept in the refrigerator to avoid any eventual temperature and lightness changes. The stability of the working solution was checked by comparing the NG main oxidation signal recorded for the fresh solution and for the same solution after 30, 60, 90 and 120 min, respectively. The peak potentials and currents did not change for at least 90 min, but signal intensity increased by 15% after 120 min; therefore, the working solution is considered stable enough to allow multiple voltammetric recordings. Despite the fact that these results emphasized that, in time, the NG solutions underwent some transformations which affected the anodic peak current; they did not allow us to conclude about the nature of these changes, and further complementary studies are necessary.

#### 3.3.2. Linear Range, Limits of Detection and Quantification

**Figure 6:**
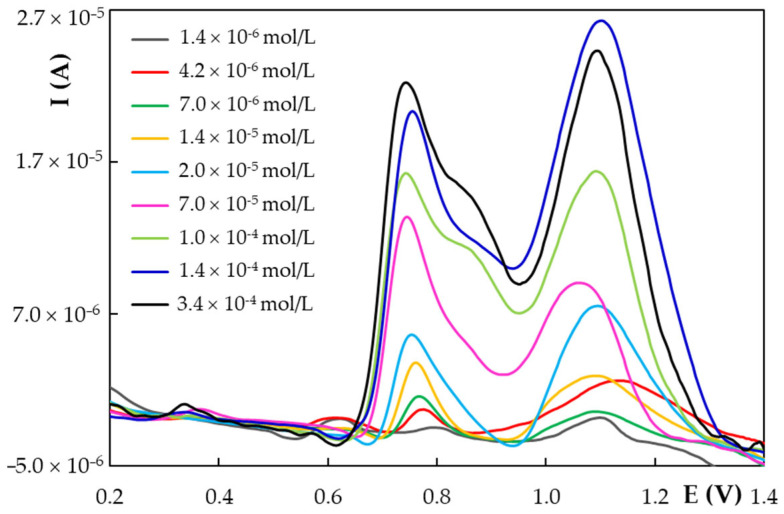
Selected DP voltammograms recorded at HB_PGE* for different NG concentrations in 0.05 mol/L KHPT pH 4.00 solution.

Due to the fact that pulse voltammetric techniques are more sensitive than the linear sweep ones, DPV was further applied for NG quantification using the previously optimized conditions, i.e., HB_PGE* as working electrode and 0.05 mol/L KHPT pH 4.00 solution as supporting electrolyte and varying the NG concentration in the range 2.00 × 10^−7^–3.40 × 10^−4^ mol/L. At NG concentrations lower than 1.40 × 10^−6^ mol/L the signal was too small to be measured with accuracy while at concentrations above 1.40 × 10^−4^ mol/L NG the current of the NG DPV oxidation peak (I_p_) situated at ~0.750 V did not further increase significantly, a flattening of the I_p_ = f(C_NG_) dependence took place (Figure S4). It was observed that the anodic signals enhanced with NG content increase (Figure 6) and I_p_ dependence on the analyte concentration presented two linear ranges expressed by the following regression equations: I_p_ (A) = 0.3684 × C_NG_ (mol/L)–3.28 × 10^–8^ (R^2^ = 0.9992) for the concentration range 1.40 × 10^−6^–2.00 × 10^−5^ mol/L NG and I_p_ (A) = 0.0843 × C_NG_ (mol/L) + 6.05 × 10^–6^ (R^2^ = 0.9856) for the concentration range 2.00 × 10^−5^–1.40 × 10^−4^ mol/L NG. The limits of detection and quantification of 6.02 × 10^−7^ mol/L NG and 1.82 × 10^−6^ mol/L NG, respectively, were assessed as 3.3 × SD/b and 10.0 × SD/b [53], respectively, where SD represents the standard deviation of the intercept and b the slope of the regression equation corresponding to the lower concentration linear range.

Starting from the previously obtained results, according to which NG main oxidation signal was a diffusion and adsorption-controlled process, the NG adsorption behavior at the HB_PGE* was exploited in order to perform NG quantification at concentrations below 1.40 × 10^−6^ mol/L by applying adsorptive stripping differential pulse voltammetry (AdSDPV). Therefore, the influence of the accumulation time (t_acc_) on NG main oxidation peak was studied in the range 1−360 s using a 7.00 × 10^−6^ mol/L NG in 0.05 mol/L KHPT pH 4.00 solution and a potential of 0.000 V applied to the HB_PGE*. The intensity of the NG investigated signal increased with the deposition time to 240 s and remained constant for longer accumulation periods (Figure S5) due to the saturation of the electrode surface with NG molecules. Thus, the optimum t_acc_ was considered to be 240 s and it was further used to observe the effect of the accumulation potential (E_acc_) on the same NG signal. The results emphasized that the variation of E_acc_ from -0.200 V to 0.200 V had no effect. Thus, using AdSDPV by applying a t_acc_ of 240 s and an E_acc_ of 0.000 V the influence of the NG concentration on its main oxidation peak was investigated. A linear dependence, described by the regression equation I_p_ (A) = 2.9410 × C_NG_ (mol/L) − 1.67 × 10^–6^ (R^2^ = 0.9995), was obtained for the concentration range 6.00 × 10^−7^–8.00 × 10^−6^ mol/L NG. The limits of detection and quantification of the AdSDPV method were 1.35 × 10^−7^ mol/L NG and 4.09 × 10^−7^ mol/L NG, respectively.

When using HB_PGE* as a working electrode, the sensitivity of the voltammetric NG detection was better by applying AdSDPV but the analysis takes six times longer in comparison to the simple DPV method. The performance characteristics of the here described methods using bare, disposable PGE were comparable with most of those previously reported in the literature for NG voltammetric determination [26,27]; there are also some with wider linear ranges [28,29,32] and lower limits of detection [28,30,32] (Table 4). This is expected when modified electrodes were employed because usually, the modifiers have catalytic effects and also generate higher effective electroactive surface areas.

#### 3.3.3. Repeatability

The precision of the DPV and AdSDPV at HB_PGE* methods developed for NG determination were assessed as repeatability and expressed as relative standard deviation (RSD%). This was evaluated by performing the measurements at three different concentration levels, situated at or near the lower, middle and upper limits of the methods’ linear range (Table 5). A new activated HB-type pencil lead was employed for each voltammetric recording. The good precision was demonstrated by the fact that all obtained RSD% values were within the accepted limits for the corresponding concentration levels [54].

### 3.4. Studies of Interferences on the Voltammetric Determination of NG at HB_PGE*

**Figure 7:**
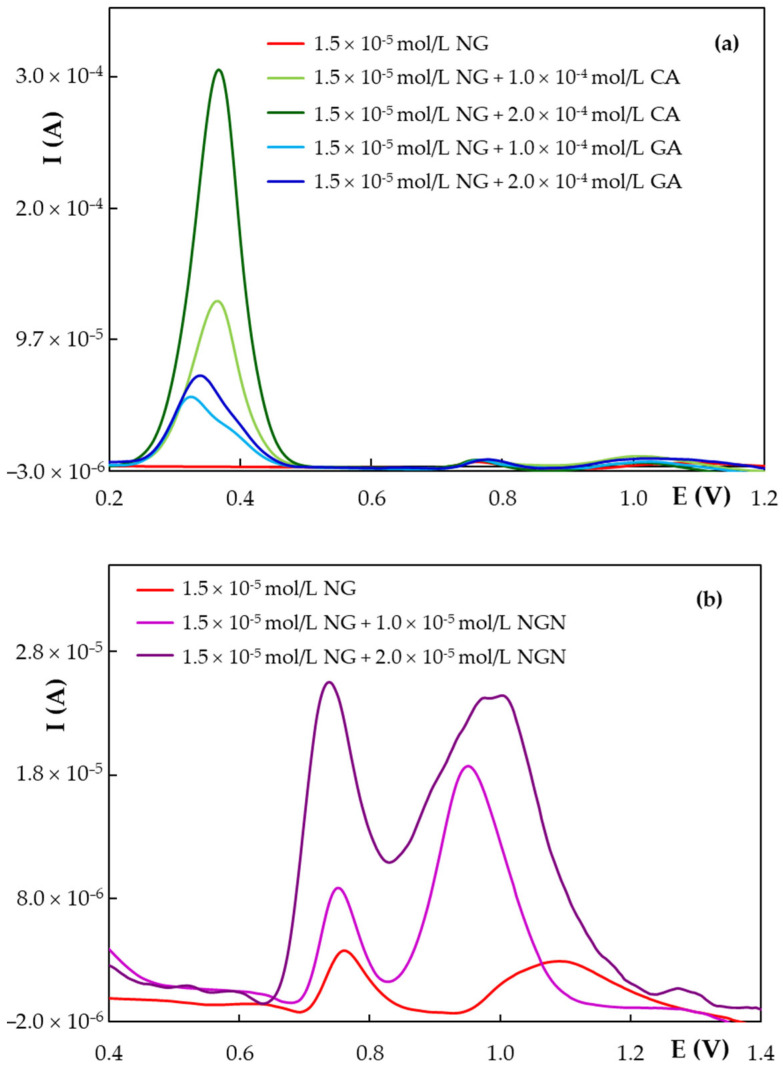
DP voltammograms recorded at HB_PGE* for 1.50 × 10^−5^ mol/L NG in 0.05 mol/L KHPT pH 4.00 solution in the presence of: (**a**) CA and GA; (**b**) NGN.

One of the weak points of the voltammetric methods at bare working electrodes is the limited selectivity in the case of structurally related compounds that have similar oxidation/reduction potentials. In plants and various foodstuffs there co-exist a large variety of polyphenols whose main electrooxidable moieties are represented by the phenolic –OH groups. The investigation of the influence of NGN, caffeic acid (CA), and gallic acid (GA) on NG DPV response at HB_PGE* emphasized that the phenolic acids, even at a tenfold higher concentration, did not interfere in the NG determination, while NGN and NG concomitant quantification in a mixture was not possible due to their very similar oxidation potentials (Figure 7). These results are explained by the fact that based on their chemical skeleton the species belonging to the two main classes of polyphenols, flavonoids (NG and NGN) and phenolic acids (CA and GA), have somewhat different electrochemical behavior and thus very different peak potentials and AOP [42]. Therefore, despite the fact that voltammetry does not offer a satisfactory species selectivity it enables the simultaneous detection of compounds from different classes. Based on the “Electrochemical index” defined by Blasco et al. [43] the developed DPV at HB_PGE* allows the quantification of intermediate AOP phenolics (e.g., bioflavonoids) in the presence of high AOP phenolics (e.g., phenolic acids).

### 3.5. Analytical Application of Voltammetric NG Determination at HB_PGE*

The applicability of the developed analytical instrument was tested to determine the content of NG by DPV at HB_PGE* in pink and white grapefruit peel and fresh juice. The analyzed samples presented an anodic peak at about 0.750 V whose intensity increased linearly with the addition of NG. Therefore, this signal was exploited for the assessment of the NG content of the investigated real samples. Validation of the electrochemical system response, when applied to real samples, was performed using HPLC-DAD-MS analysis for the same sample (Figure S6). It can be observed (Table 6) that the results obtained by DPV are somewhat higher than those resulting from HPLC analysis. This fact is not surprising because, as already mentioned before, DPV cannot differentiate between compounds with very similar structures and similar oxidation potentials (e.g., NG and its metabolite NGN) as is the case of those belonging to the bioflavonoid class which, based on their oxidation potential, are considered compounds with intermediate AOP. The NGN detected in the analyzed real samples was at a very low concentration level (Table 6) and was even not detected in the pink variety during the analysis of the fresh juice. These results proved that, probably, it was not sufficient time to allow the transformation of NG in its aglycon, NGN. Moreover, this proved the lack of DPV discrimination between compounds with analogous structures.

**Figure 8:**
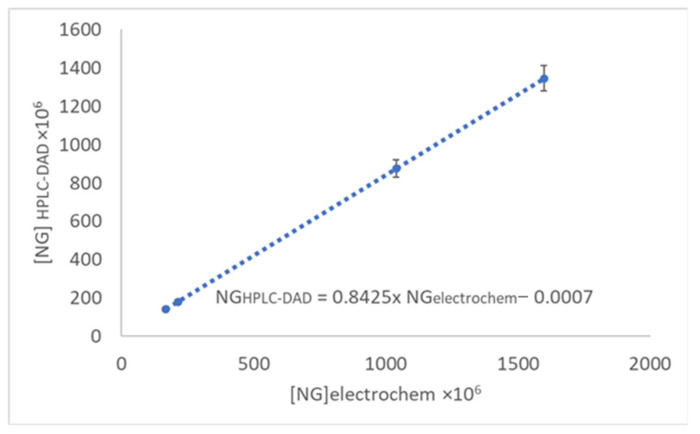
Correspondence between NG analysis by DPV at HB_PGE* and HPLC-DAD in real samples.

Electrochemical data obtained for real samples are matching to those obtained by chromatographic analysis, as could be noticed from Figure 8, thus being proved the suitability of using the developed electrochemical system for the assessment of TCP with intermediate AOP expressed as NG equivalent.

## 4. Conclusions

The present work reports for the first time the electrochemical behavior of NG at the cheap pencil graphite electrode which was previously potentiostatically activated (PGE*). At this single-use electrode, NG is irreversibly oxidized with one electron and one proton, in a pH-dependent, mixed (diffusion and adsorption-controlled) electrode process. Exploiting NG main oxidation signal at HB_PGE*, situated at ~0.750 V (in 0.05 mol/L KHPT pH 4.00), DPV and AdSDPV methods were developed for the bioflavonoid quantification with LoDs at submicromolar level (6.02 × 10^−7^ and 1.35 × 10^−7^ mol/L NG, respectively). The applicability of the developed electrochemical method was assessed by estimating the NG content of grapefruit peel and fresh juice. The results obtained by DPV at HB_PGE* were higher than those given by HPLC-DAD-MS analysis but they correlated well. This observation can be explained by the fact that voltammetry does not offer a satisfactory selectivity for species with similar structures, but it can be successfully used to appraise the total amount of compounds with close oxidation potentials, e.g., analytes with similar AOP. Therefore, the developed electrochemical system proved to be suitable as a rapid and simple method for the screening of the TCP with intermediate AOP in citrus samples.

## Figures and Tables

**Table 1:** The main DPV anodic peak (E_p_~0.800 V) currents recorded for 1.50 × 10^−5^ mol/L NG in ABS pH 4.00 at Rotring HB type PGE bare and electroactivated in different conditions.

	Electroactivation Technique	CV (−0.500 to 2.000 V; n = 10; v = 0.500 V/s)	E = ct. (2.000 V; 60 s)	None
Medium		I_p_ (A)		
BRB pH 2.21		2.42 × 10^−6^	4.47 × 10^−6^	4.70 × 10^−6^
ABS pH 4.00		4.78 × 10^−6^	3.75 × 10^−6^	
PBS pH 7.00		4.84 × 10^−6^	6.33 × 10^−6^	
M NaOH		6.34 × 10^−6^	4.85 × 10^−6^	

E_p_ and I_p_: potential (V) and current (A) of NG main oxidation peak situated at ~0.800 V; n: number of voltammetric cycles; v: scan rate (V/s).

**Table 2:** The regression equations describing the peak potential (E_p_, V) vs. pH dependencies of the NG main oxidation signal obtained at HB_PGE* in BRB solutions.

Technique	Regression Equation	x/n
CV	E_p_ = −0.055 × pH + 1.160 (R^2^ = 0.9848)	0.93
DPV	E_p_ = −0.050 × pH + 1.046 (R^2^ = 0.9848)	0.85

R^2^—determination coefficient.

**Table 3:** The regression equations describing the different peak currents (I_p_, A) vs. scan rate (v, V/s) and E_p_ = f(log v) dependencies of the NG main oxidation signal obtained at HB_PGE* corresponding to the cyclic voltammograms from Figure 4.

I_p_ = f(v)	I_p_ = f(v^1/2^)	log I_p_ = f(log v)	E_p_ = f(log v)	E_p_ = f(v)
I_p_ = 1.00 ×10^−4^ × v + 2.00 × 10^−6^ R^2^ = 0.9838	I_p_ = 7.00 × 10^−5^ × v^1/2^ − 8.00 × 10^−6^ R^2^ = 0.9899	log I_p_ = 0.8345 × log v − 4.0614 R^2^ = 0.9790	log E_p_ = 0.0938 × log v + 0.9204 R^2^ = 0.9806	E_p_ = 0.7722 × v + 0.7544 R^2^ = 0.9768

**Table 4:** The performance characteristics of the electrochemical detection methods reported in the literature for naringin determination.

Technique	Electrode	Linear Range (mol/L)	Limit of Detection (mol/L)	Sample	Ref.
DPV	Poly-o-aminophenol MIP/graphite electrode	6.00 × 10^−5^–1.40 × 10^−4^	1.60 × 10^−5^		[27]
1-DLSV	SDS in situ MWCNTs-COOH/GCE	7.50 × 10^−7^–1.00 × 10^−4^	1.40 × 10^−7^	Grapefruit juice	[26]
DPV	Polyaluminon/f-SWCNTs/GCE	1.00 × 10^−7^–2.50 × 10^−5^	2.00 × 10^−8^	Orange and grapefruit juice	[8]
DPV	PolyEA/MWCNTs/GCE	5.00 × 10^−8^–1.00 × 10^−4^	1.40 × 10^−9^	Grapefruit juice	[28]
DPV	dsDNA/PDDA-MWCNTs/PGE	1.00 × 10^−7^–1.00 × 10^−3^	1.72 × 10^−8^	Orange, lemon, grapefruit juice	[29]
CSDPV	HMDE	1.72 × 10^−7^–6.88 × 10^−5^	5.50 × 10^−8^	Grapefruit juice	[30]
Flow injection/ AdSDPV	Nujol based CPE	-	d: 1.40 × 10^−8^ m.e: 9.00 × 10^−9^	-	[31]
	Diphenylether based CPE		d: 1.90 × 10^−8^ m.e: 1.70 × 10^−8^		
PE (Amp)	CPPI-TiO_2_/CdS/FTO	1.00 × 10^−6^–3.32 × 10^−4^	3.00 × 10^−8^	Orange, limon, tangerine juice	[6]
PE (CV)	Au NRs/g-C_3_N_4_/Cys/GCE	1.00 × 10^−10^–1.00 × 10^−4^	3.00 × 10^−11^		[32]
DPV	PGE	1.40 × 10^−6^–2.00 × 10^−5^ 2.00 × 10^−5^–1.40 × 10^−4^	6.02 × 10^−7^	Grapefruit peel and fresh juice	This work
AdSDPV		6.00 × 10^−7^–8.00 × 10^−6^	1.35 × 10^−7^		

MIP: molecularly imprinted polymer; 1-DLSV: first-order derivative linear sweep voltammetry; SDS: sodium dodecylsulphate; GCE: glassy carbon electrode; MWCNTs: multi-walled carbon nanotubes; f-SWCNTs: functionalized single-walled carbon nanotubes; polyEA: electropolymerized ellagic acid; PGE: pencil graphite electrode; dsDNA: double-stranded DNA; PDDA: polydialyldimethylammonium chloride; CSDPV: cathodic stripping differential pulse voltammetry; HMDE: hanging mercury drop electrode; CPE: carbon paste electrode; d: direct; m.e.: medium exchange; PE: photoelectrochemistry; Amp: amperometry; FTO: fluorine doped tin oxide electrode; CPPI: chloroprotoporphyrin IX iron(III); Au NRs: gold nanorods; g-C_3_N_4_: graphitic carbon nitride nanosheets; Cys: cysteine.

**Table 5:** Results obtained in the repeatability study for NG determination.

Technique	AdSDPV			DPV		
NG concentration (mol/L)	6.00 × 10^−7^	2.00 × 10^−6^	8.00 × 10^−6^	2.00 × 10^−6^	8.00 × 10^−6^	5.00 × 10^−5^
RSD%	9.32	4.52	2.12	7.62	5.47	2.13

**Table 6:** NG concentration in grapefruit fresh juice and peel assessed using DPV at HB-PGE* and comparative HPLC-DAD-MS analysis, respectively.

Sample		NG Concentration (mol/L)		NGN Concentration (mol/L)
		DPV at HB_PGE*	HPLC-DAD-MS	HPLC-DAD-MS
Pink grapefruit	Peel	1038.68 × 10^−6^	875.05 × 10^−6^	7.50 × 10^−7^
	Fresh juice	214.05 × 10^−6^	180.33 × 10^−6^	N.D.
White grapefruit	Peel	1597.18 × 10^−6^	1345.56 × 10^−6^	5.16 × 10^−7^
	Fresh juice	166.73 × 10^−6^	140.46 × 10^−6^	4.41 × 10^−7^

N.D.—Not detected

## Data Availability

Data is contained within the article and supplementary material.

## Supplementary Materials

The following supporting information can be downloaded at: https://www.mdpi.com/article/10.3390/antiox11122306/s1, Figure S1: Selected cyclic voltammograms recorded at HB_PGE* for 2.50 × 10^−4^ mol/L NG in BRB solutions with different pH values; Figure S2: The variation of DPV anodic peak current recorded at HB_PGE* for a 1.50 × 10^−5^ mol/L NG solution depending on the nature of the supporting electrolytes; Figure S3: The variation of DPV anodic peak current recorded at HB_PGE* for 1.50 × 10^−5^ mol/L NG with the KHPT concentration in solutions with pH 4.00; Figure S4: The variation of DPV anodic peak current recorded at HB_PGE* with NG concentration in 0.05 mol/L KHPT pH 4.00 solution; Figure S5: The influence of the accumulation time (t_acc_) on NG main anodic peak current recorded by DPV at HB_PGE* for a 7.00 × 10^−6^ mol/L NG in 0.05 mol/L KHPT pH 4.00 solution; E_acc_ = 0.000 V; Figure S6: HPLC-DAD-MS chromatogram for sample of fresh juice from white grapefruit.
