# Supplementary material for: Electroanalysis of Naringin at Electroactivated Pencil Graphite Electrode for the Assessment of Polyphenolics with Intermediate Antioxidant Power
Source: Antioxidants (Basel). 2022 Nov 22;11(12):2306. doi: 10.3390/antiox11122306 (PMC9774430; doi:10.3390/antiox11122306)
Supplement: Supplementary file 1 [file antioxidants-11-02306-s001.zip › antioxidants-2032634-supplementary.pdf]

Supplementary information

Electroanalysis of Naringin at Electroactivated Pencil Graphite Electrode for the Assessment of Polyphenolics with Intermediate Antioxidant Power

Iulia Gabriela David [1,*], Simona Carmen Litescu [2,*], Raluca Moraru [1], Camelia Albu [2], Mihaela Buleandra [1],
Dana Elena Popa [1], Sorin Riga [3], Adela Magdalena Ciobanu [4,5] and Hassan Noor [6]

[1] Department of Analytical Chemistry and Physical Chemistry, Faculty of Chemistry, University of Bucharest, Panduri Av. 90-92, District 5, 050663 Bucharest, Romania
[2] National Institute of Research and Development for Biological Sciences, 296 Independenței Bd., District 6, 060031 Bucharest, Romania;
[3] Department of Stress Research & Prophylaxis "Prof. Dr. Al. Obregia" Clinical Hospital of Psychiatry, Berceni Av. 10, District 4, 041914 Bucharest, Romania
[4] Department of Psychiatry "Prof. Dr. Al. Obregia" Clinical Hospital of Psychiatry, Berceni Av. 10, District 4, 041914 Bucharest, Romania
[5] Discipline of Psychiatry, Neurosciences Department, Faculty of Medicine, "Carol Davila" University of Medicine and Pharmacy, Dionisie Lupu Street 37, 020021 Bucharest, Romania
[6] Department of Surgery, Faculty of Medicine, "Lucian Blaga" University Sibiu, Lucian Blaga Street 25, 550169 Sibiu, Romania
* Correspondence: gabrielaiulia.david@g.unibuc.ro (I.G.D.); slitescu@gmail.com (S.C.L.)

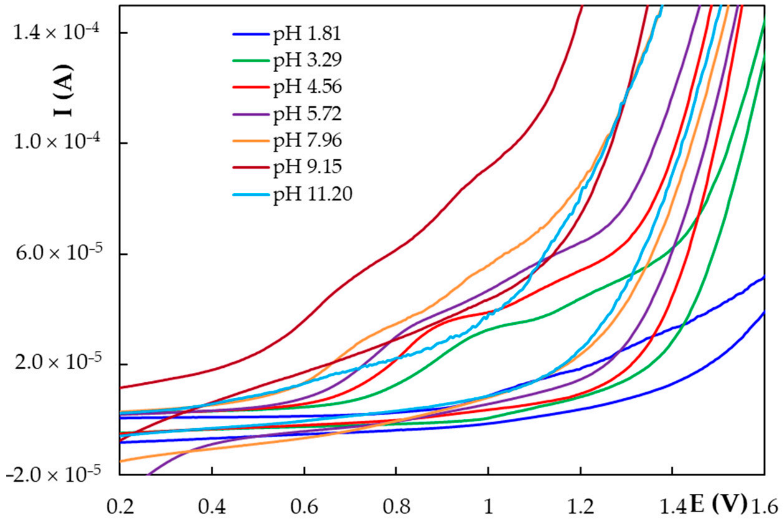

Figure S1. Selected cyclic voltammograms recorded at HB_PGE* for 2.50 × 10^{-4} mol/L NG in BRB solutions with different pH values.

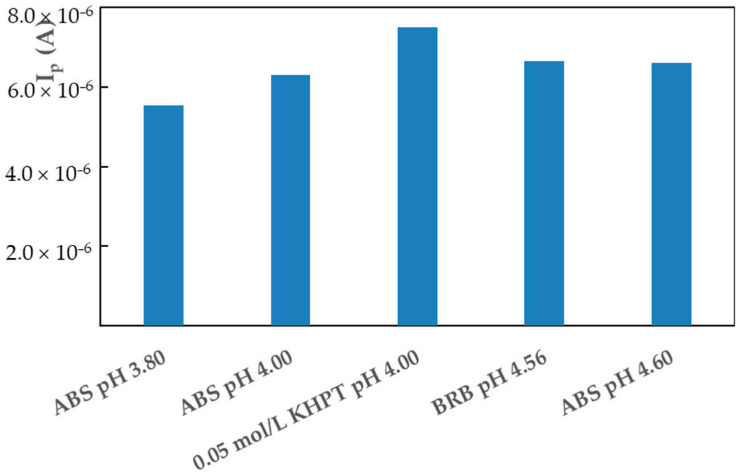

Figure S2. The variation of DPV anodic peak current recorded at HB_PGE* for a 1.50×10^{-5} mol/L NG solution depending on the nature of the supporting electrolytes.

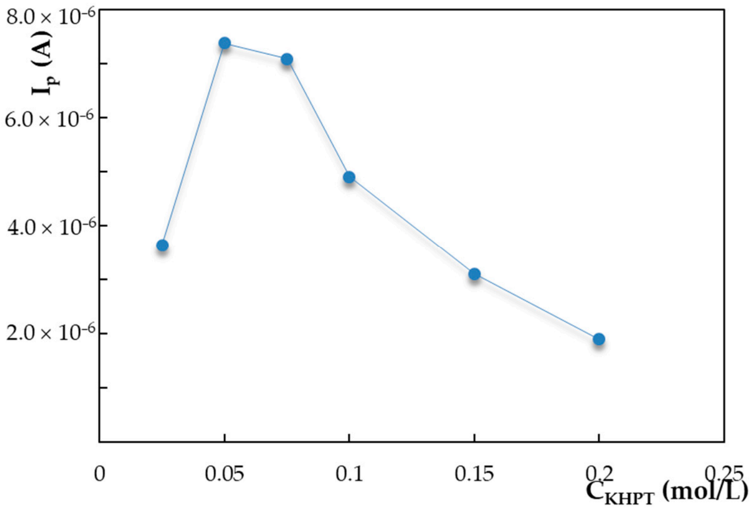

Figure S3. The variation of DPV anodic peak current recorded at HB_PGE* for 1.50×10^{-5} mol/L NG with the KHPT concentration in solutions with pH 4.00.

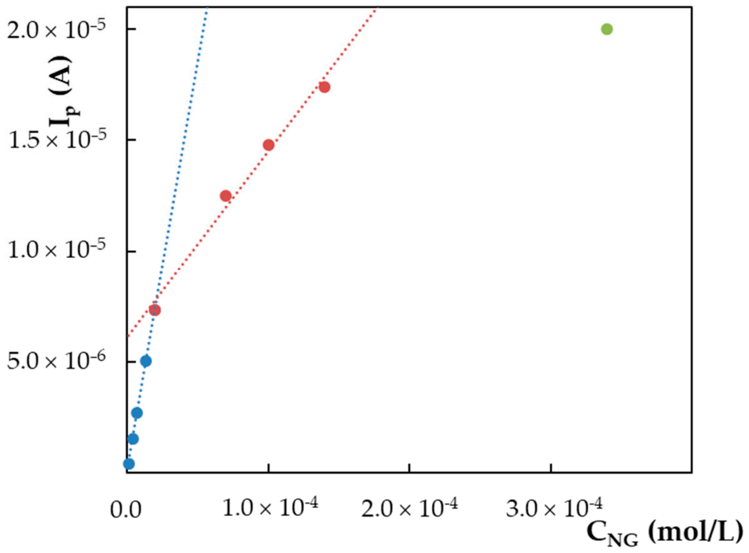

Figure S4. The variation of DPV anodic peak current recorded at HB_PGE* with NG concentration in 0.05 mol/L KHPT pH 4.00 solution.

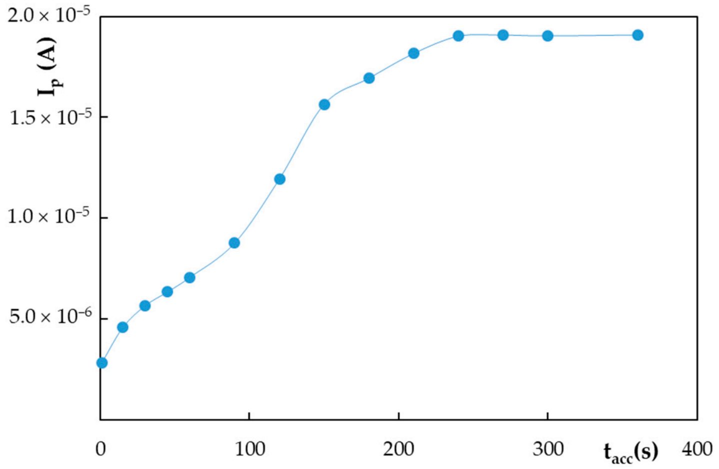

Figure S5. The influence of the accumulation time (t_{acc}) on NG main anodic peak current recorded by DPV at HB_PGE* for a 7.00 × 10^{-6} mol/L NG in 0.05 mol/L KHPT pH 4.00 solution; E_{acc} = 0.000 V.

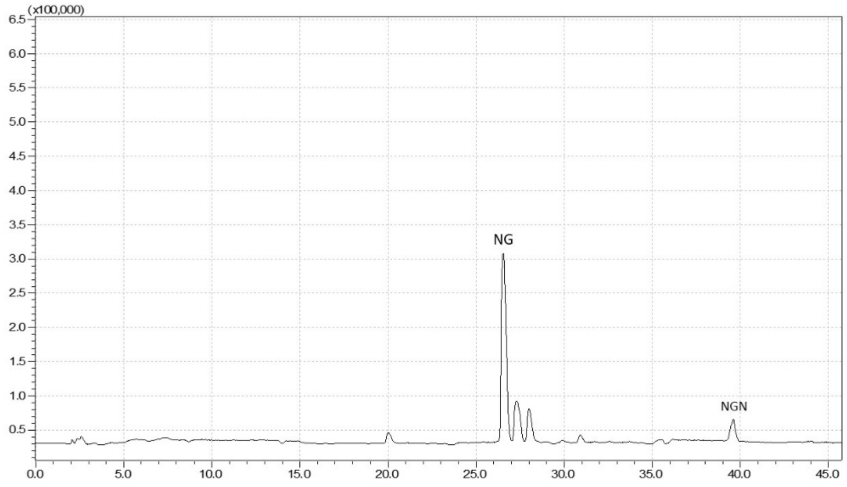

Figure S6. HPLC-DAD-MS chromatogram for sample of fresh juice from white grapefruit
